# Appraisal of work ability in relation to job-specific health requirements in ambulance workers

**DOI:** 10.1007/s00420-016-1181-z

**Published:** 2016-11-02

**Authors:** A. van Schaaijk, J. S. Boschman, M. H. W. Frings-Dresen, J. K. Sluiter

**Affiliations:** Coronel Institute of Occupational Health, Academic Medical Center, University of Amsterdam, PO Box 22700, 1100 DE Amsterdam, The Netherlands

**Keywords:** Work ability, Ambulance, Job-specific, Workers Health Surveillance (WHS), Job-specific requirements

## Abstract

**Purpose:**

To gain insight into which job-specific health requirements relate to work ability, the following two research questions were formulated: *Which job*-*specific health requirements are associated with the appraisal of work ability in ambulance drivers and paramedics? How are appraisals of physical and mental work ability associated with the appraisal of overall work ability in ambulance drivers and paramedics?*

**Method:**

Workers Health Surveillance cross-sectional data of 506 ambulance workers (236 drivers and 270 paramedics) were used. The tests for specific job requirements were divided into six categories. Work ability was appraised as overall, physical and mental/emotional. Multiple linear stepwise regression analyses were used to model the associations.

**Results:**

Outcomes in ‘raised alertness and judgment ability’ (*R* ^2^ = 0.09), ‘job-specific physical abilities’ (*R* ^2^ = 0.10) and ‘emotional peak load’ (*R* ^2^ = 0.07) significantly explained appraised overall, physical and mental/emotional work ability. Physical and mental/emotional work ability together explained 48.3% of the variance of overall work ability. The explained variance by physical and mental/emotional work ability was almost 4% higher in drivers than in paramedics.

**Conclusions:**

Overall work ability was significantly explained by outcomes in ‘raised alertness and judgment ability’ and ‘emotional peak load.’ Physical work ability was significantly explained by ‘job-specific physical abilities’ and ‘raised alertness and judgment ability’ outcomes, while ‘emotional peak load’ and ‘raised alertness and judgment ability’ outcomes significantly explained mental/emotional work ability. Physical and mental/emotional work ability explains the same proportion of variance in overall work ability.

## Introduction

Work ability in ambulance workers is endangered due to the high physical and mental/emotional demands placed on them. The attribution to appraised work ability by job-specific health requirements has not been determined in ambulance workers. Evidence shows that ambulance workers have an injury rate of 3–5 times higher than the general working population (Maguire and Smith 2013; Roberts et al. 2015), and a higher health risk (Boreham et al. 1994; Hansen et al. 2012; Sterud et al. 2006). The risk for musculoskeletal disorders in the lower back is 13 times higher than for health professionals, while the risk for mental disorders is almost 14 times higher (Roberts et al. 2015). Ambulance work endangers the physical and mental health of ambulance workers and demands adequate health.

Consequently, it is clear that monitoring the health and work ability of ambulance workers is important to prevent deterioration of health and prevent hazardous situations. A strategy that can be used to monitor the health of ambulance workers is a job-specific Workers Health Surveillance (WHS) (Sluiter 2006; Sluiter et al. 2013).

The construct of work ability has been defined over the years by Ilmarinen et al. (1997) as ‘the quality of workers at present and in the near future to be able to do their job satisfactorily with respect to work demands, health and mental resources.’ Work ability is especially important in jobs with specific requirements because of the high job demands. Ambulance work is such an occupation, with both high mental and physical demands (Boreham et al. 1994; Pek et al. 2015; Roberts et al. 2015) and a higher risk for lower work ability compared to other health professionals (Klasan et al. 2013; Roberts et al. 2015; Sterud et al. 2006; van den Berg et al. 2009).

Specific psychological demands are being able to drive with flashing lights and sirens, working under time pressure with critical and sometimes aggressive bystanders when human life is at stake, and making complicated (medical) decisions involving a person’s health in perilous situations (Sluiter 2006). Besides psychological health demands, the psychosocial health demands are an important part of work ability. The emotional demands that accompany with an emergency call and coping with traumatic experiences lead to stress reactions (Sluiter et al. 2003) and may result in post-traumatic complaints and depression (Sterud et al. 2006; van der Ploeg and Kleber 2003). Under demanding circumstances, the ambulance worker needs to remain a professional and must be able to make rational decisions. The work of ambulance workers is also physically strenuous. Ambulance workers need to be able to maintain a certain position for a period of time to be able to treat a patient. Their physique is also challenged when they need to take challenging or unconventional routes to reach a victim or patient.

To monitor all these specific requirements of ambulance workers, a job-specific WHS has been developed in the Netherlands in collaboration with the ambulance sector organization (Sluiter and Frings-Dresen 2005). The WHS screens on physical capacity, work-related fatigue, sleepiness, post-traumatic stress complaints and complaints of depression and anxiety. These are categorized as ‘the ability to perceive and communicate,’ ‘be able to have raised alertness and judgment ability,’ ‘be capable of handling emotional peak load,’ ‘job-specific physical abilities,’ and ‘having respiratory complaints’ or ‘having skin complaints’ that affect working. In addition to these categories, the appraised overall, physical and mental/emotional work ability was scored. It is still unknown whether these health requirements are associated with the appraisal of work ability.

To satisfactorily function in work and guarantee a high quality of healthcare, it is important that ambulance workers comply to minimum standards. Although the job requirements for drivers and paramedics are similar, it is not known how health complaints or problems in work functioning relate to work ability in these different occupations. In the Netherlands in 2012, 2218 ambulance paramedics (1445 male, 773 female) and 1949 ambulance drivers (1712 male, 237 female) were employed who carried out 1,100,419 rides, of which 500,835 were emergency calls (Boers et al. 2013).

To date, no study has gathered information on job-specific aspects of health and work ability so specifically. Work ability can be divided into two aspects: mental and physical. Although it is known that mental and physical health has a different impact on self-reported work ability (van de Vijfeijke et al. 2013), it is still unclear how the self-reported mental and physical work ability relates to the appraisal of overall work ability. According to a study of Klasan et al. (2013), the aspect that predicts low work ability in ambulance workers best is the amount of strenuous physical activity during work. But which job-specific health complaints greatly reduce work ability? It is still unknown where interventions should be aimed at, to expect the greatest positive change in work ability.

To explore the composition of appraised work ability and to study whether work ability appraisal has an additional value in the WHS, this study assesses the explained variance of job-specific mental and physical health requirements and ambulance workers’ appraisal of their own current work ability. This leads to the following research question: *Which job*-*specific health requirements are associated with the appraisal of work ability in ambulance drivers and paramedics?*

We expect physical job requirements to have a bigger impact on self-reported work ability than the mental requirements and will show up as more important in the model. We expect the highest explained variance of appraised overall work ability to be physical work ability for both occupations, based on previous studies in ambulance workers and other occupations (Klasan et al. 2013; van de Vijfeijke et al. 2013). However, we expect that the mental demands are more important for ambulance drivers than for paramedics, since raised alertness is more frequently demanded for drivers. The second research question is: *How are appraisals of physical and mental work ability associated with the appraisal of overall work ability in ambulance drivers and paramedics?*

## Methods

We used Dutch WHS data collected in the ambulance sector over the period October 20, 2011 until January 22, 2013. Cross-sectional data of the WHS among 506 ambulance paramedics (*n* = 270) and drivers (*n* = 236) were available for analyses. All employees participated on a voluntary basis. The WHS was conducted according to a national protocol, ensuring the safety and anonymity of the participants. An occupational physician and/or assistant conducted all tests. The collective labor agreement of the ambulance sector 2011–2013 included an agreement which stated that the data of the WHS were stored in a national database and that data were to be analyzed anonymously by the Academic Medical Center (AMC) for further research (AZN 2011). Ambulance workers were reminded of this agreement in writing before participating in the WHS. The Medical Ethical Commission of the AMC authorized this study and decided that a full application was not required.

Specific job requirements are considered important requirements to be met by ambulance workers to satisfactorily fulfill their work and cope with their demanding working conditions without impairing their own health or the safety of others. Important requirements for ambulance work that can affect work ability were distinguished in six categories: ‘to perceive and communicate’; ‘having raised alertness and judgment ability’; ‘dealing with emotional peak load’; ‘job-specific physical abilities’; ‘respiratory complaints’; and ‘skin complaints.’

To assess ‘the ability to perceive and communicate,’ hearing and vision tests were performed. Necessary eyesight for perception consisted of color perception and acuity tests. Physicians tested color perception with the Ishihara test for color blindness (Thiadens et al. 2013). The Landolt C-ring test was used to test visual acuity. Three yes/no questions on eyesight were about whether the employee had problems with vision during work, at night and with reading. The whisper-speech test tested the ability to hear human speech in conversation (Eekhof et al. 1997). Two signal questions were asked about problems with the ability to hear inside and outside the ambulance.

‘Raised alertness and judgment ability’ were assessed by measuring the magnitude of work-related fatigue, mental complaints, alertness and sleepiness. Work-related fatigue was measured by the need for recovery after working time scale (NFR) (Veldhoven and Meijman 1994). Mental complaints were measured using the Brief Symptoms Inventory (BSI) subscale of depression. Alertness and sleepiness were measured using the Epworth Sleepiness Scale (ESS) (Johns 1991). Five questions on having problems concerning thinking, fatigue, depression, alertness and sleep complaints were included in yes/no items. Examples of signal questions are: ‘Have you had difficulty in determining what successive sub-activities you had to perform on a patient recently?’ and ‘Would you consider yourself as someone with sleeping complaints?’

The third group of requirements addressed health complaints related to ‘the ability to handle emotional peak load’ and were addressed using the Impact of Event Scale to assess the level of post-traumatic complaints (van der Ploeg et al. 2004). Anxiety level was addressed through the BSI subscale of anxiety (Derogatis and Spencer 1993; Meijer et al. 2011). Five signal questions on traumatic experiences were included in this category. Limitations due to traumatic experiences and experienced emotional load and aggression were asked about in yes/no items, e.g., ‘Do you encounter any limitations in your work functioning due to severe traumatic events that you experienced recently?’ and ‘Did you experience any aggression during your work toward you or a colleague recently?’

‘Job-specific physical abilities’ were assessed through physical performance tests and four signal questions to assess musculoskeletal complaints. The ability to lift, maintain balance and grip strength was measured using an ambulance balance test (Punakallio 2003). The ability to work in a kneeled position was assessed by means of a manual cardiopulmonary resuscitation (CPR) test on a dummy on floor level (Coronel Institute of Occupational Health 2013). The ability to clamber and climb, lift, maintain balance, grip strength and energetic peak load was measured in the ambulance stair climb test (Plat et al. 2010; Teh and Aziz 2000). Signal questions for limitations in physical functioning were asked concerning physical complaints, e.g., ‘Have you encountered any complaints in joint or muscle which resulted in limitations in your work during the last month?’

Both ‘skin complaints’ and ‘respiratory complaints’ were assessed with one signal question each. The questions were: ‘Did you experience any needle stick or bite accidents in the past 5 years?’ and ‘Did you experience any breathing or airway complaints after incidental or repeated exposure to a high concentration of inhaled dust, fumes, gasses, or vapor in the last 6 months?’

The appraisal of overall work ability, physical work ability and mental/emotional work ability were assessed by means of one question each (El Fassi et al. 2013). Workers had to assign a number between zero (not able to do ambulance work) and ten (the best work ability ever experienced for ambulance work) to their current ability to perform ambulance work, compared to their best ever (van den Berg et al. 2009). These appraisals of work ability were used as dependent variables in the statistical analyses.

In addition to the above-mentioned categories that possibly relate to appraised work ability, personal data were included in the WHS. Occupation and gender were taken into account, being tested for significant differences. If significant differences were found, we corrected the models for this demographic and added it to each model.

Individual scores on all questions and tests were converted to a score for that specific part of the WHS as described above. Higher scores on the independent variables were indicative of worse performance or more health complaints or problems, and thus a theoretical negative effect on work ability. All missing values were on the dichotomous variables (3%) and have been replaced by zero (non-case). In all six categories, signal questions were asked with a yes or no outcome. If one of the signal questions per category or sub-category was answered with yes, the variable for this subject was categorized as a sign of reduced work ability and scored with a one. The pass or fail score on the Landolt C-ring test was determined, as all scores on three distances (with both eyes) had to be sufficient (≥0.8) to pass the test (Sluiter and Frings-Dresen 2005). A dichotomous variable was created for the Ishihara color test (Cole 2007). To pass the whisper-speech test, no more than three mistakes were allowed per ear (Eekhof et al. 1997). Need for recovery, BSI subscale scores, the ESS and the Impact of Event Scale were registered as sum score (Johns 1991; Meijer et al. 2011; van der Ploeg et al. 2004; Veldhoven and Meijman 1994). In the job-specific physical abilities category, to pass the balance test, (where an ambulance worker needed to walk over a wooden plank with an ambulance case in each hand,) the ambulance workers’ three best times out of five needed to be within 30 s. Those three best results still needed to be within 30 s when the penalty seconds were added. No more than six mistakes were allowed in total in these three fastest tests combined. The CPR test had to be completed without having to stop or get up. In the stair climb test, the end heart rate was measured using a Polar heart rate monitor and registered at the last step. The test criteria were not met when the test was not completed within 90 s, or the heart rate stayed below 85% of heart rate maximum (calculated as 220—age), unless the test was completed within 60 s. If the criteria of any test were not met, the test was scored as one and regarded as determinant of reduced work ability.

The normality of the scores on the three indicators for work ability appraisal questions (dependent variables) was checked by means of a visual inspection of the histogram graphs. First, differences between drivers and paramedics, and men and women on appraised work ability were tested to verify which demographic variables the analysis should be controlled for. Second, to assess *which job*-*specific health requirement categories are associated with appraisals of work ability*, six (multiple) blockwise linear regression analyses were performed per dependent variable to assess the explained variance per category. All variables of one of the six requirement categories were entered simultaneously in the model in order to be able to make a distinction between categories and their impact on appraised work ability. A model for overall work ability was created where the category with the highest explained variance was entered first, followed by the next highest, and so on, until adding a category no longer led to significant change in explained variance. The final model was based on the previous model and included a correction for gender.

To answer the *second research question* and address the contributions of physical and mental work ability to the overall appraised work ability, a linear regression was performed with overall work ability as dependent variable and the appraisal of physical and mental work ability as independent variables. As a final check, each model in the above analyses was tested for heteroscedasticity (through a visual inspection of the residual plots) and collinearity (through collinearity statistics and VIF score). A VIF score over 2.5 was set as cause for concern of collinearity.

All statistical analyses were performed using the IBM SPSS Statistics 22 software.

## Results

Of all 509 ambulance workers that participated in the WHS, the data of 506 could be used for statistical analyses. Of the drivers, 205 were male and 31 were female, and of the paramedics, 196 were male and 74 were female. Three percent of 11,638 values were missing. Sample demographics are shown in Table 1. The average appraised of overall, mental and physical appraised work ability was, respectively, 8.5 (SD = 0.88), 8.5 (SD = 1.04) and 8.7 (SD = 0.91). There was no reason to assume that heteroscedasticity or collinearity occurred in any of the tests.

**Table 1:** Occupation, gender and age of ambulance workers (*n* = 506)

	Mean	SD	*N*	%
Occupation				
Driver			236	47
Paramedic			270	53
Gender				
Men			401	79
Women			105	21
Age (years)	43.1	8.6		

The number of cases and the sum scores on the dependent and independent variables are shown in Table 2 for descriptive purposes.

**Table 2:** Outcomes [in mean (+SD) or cases (*n*%)] of 506 ambulance workers in appraised work ability and health requirements in six categories

		Mean	SD	Cases n*	Cases %*
*Dependent variables*					
Overall work ability		8.5	0.88	0	0.0
Physical work ability		8.5	1.04	5	1.0
Mental/emotional work ability		8.7	0.91	1	0.2
*Categories of independent variables*					
1.					
Vision problems				81	16.0
Hearing problems				36	7.1
Acuity problems				47	9.3
Color vision problems				38	7.5
Hearing human speech problems				16	3.2
2.					
Raised alertness and judgment complaints				62	12.3
Work-related fatigue (0–11)		0.6	1.26	7	1.4
Symptoms of depression (0–24)		0.5	1.15	36	7.1
Alertness and sleepiness problems (0–24)		2.9	2.31	4	0.8
3.					
Limitations due traumatic events				102	20.2
Symptoms of PTSD (0–75)		3.9	6.21	15	3.0
Symptoms of anxiety disorder (0–24)		0.7	1.13	38	7.5
4.					
Musculoskeletal complaints				107	21.1
Reduced balance				10	2.0
Reduced manual CPR abilities				20	4.0
Reduced stair climb abilities				100	19.8
5.					
Respiratory complaints				6	1.2
6.					
Skin complaints				11	2.2

* Based on the cutoff values in the WHS (Coronel Institute of Occupational Health 2013)

No statistically significant differences in the three work ability appraisals were found between ambulance drivers and paramedics (*p* values of 0.18, 0.72 and 0.81). Gender explained a significant part of variance of appraised work ability (*p* ≤ 0.001) and was therefore controlled for in the later models. The category that significantly predicts overall work ability most was ‘raised alertness and judgment ability’ in both occupations. When corrected for gender, 10.4% of the variance in overall work ability was explained. When comparing occupations, ‘raised alertness and judgment ability’ explained significant variability in both occupations, with 16% for ambulance drivers and an 8% for paramedics. The categories in order of explained variance are shown in Table 3.

**Table 3:** Order of explained variance per job requirement category per dependent variable from highest to lowest

	Overall work ability	Physical work ability	Mental work ability
1	Raised alertness and judgment ability*	Job-specific physical abilities*	Emotional peak load*
2	Emotional peak load*	Raised alertness and judgment ability*	Raised alertness and judgment ability**
3^n.s.^	Job-specific physical abilities	Emotional peak load	Job-specific physical abilities
4^n.s.^	Perceive and communicate	Perceive and communicate	Respiratory complaints
5^n.s.^	Skin complaints	Respiratory complaints	Perceive and communicate
6^n.s.^	Respiratory complaints	Skin complaints	Skin complaints

* Significant improvement in model indicated by *F*-change at *p* < 0.01

** Significant improvement in model indicated by *F*-change at *p* < 0.05

^n.s.^No significant improvement

The categories of ‘gender,’ ‘raised alertness and judgment ability’ and ‘emotional peak load’ were added to build the final model that explained 12.2% of the variance of the appraised overall work ability.

‘Job-specific physical abilities’ were the highest contributing category for physical work ability after correction for gender (*R* ^2^ = 0.13, *p* ≤ 0.001). The model that included ‘gender,’ ‘job-specific physical abilities’ and ‘raised alertness and judgment ability’ explained 15.3% of the variance of the appraised physical work ability. There were no differences between ambulance drivers and paramedics.

‘Emotional peak load’ was the highest contributing category of health requirements for mental work ability when corrected for gender (*R* ^2^ = 0.09, *p* ≤ 0.001). The final model contained the categories of ‘gender,’ ‘emotional peak load’ and ‘raised alertness and judgment ability’ and explained 11.0% of the variance of the appraised mental/emotional work ability. In ambulance drivers, to explain variability mental/emotional work ability, the most important category was ‘raised alertness and judgment ability’ followed by ‘job-specific physical abilities’ and ‘emotional peak load.’ In ambulance paramedics, a different sequence of importance was observed. The most important category for paramedics was ‘emotional peak load’ followed by ‘raised alertness and judgment ability’ and ‘skin complaints.’

For the second question, appraised physical work ability explained a statistically significant proportion of 37.4% of the variance on the total score of overall work ability, while appraised mental work ability explained a statistical significant proportion of 39.1% (Table 4). Both variables together explained 48.3% of the total variance on overall work ability appraisal. The regression model for overall work ability for drivers had an explained variance of 0.51: *Y* = 2.198 + (−0.141 ‘Gender’) + (0.310 ‘Physical work ability’) + (0.412 ‘Mental/emotional work ability’). For paramedics, the model for overall work ability had an explained variance of 0.47: *Y* = 2.584 + (−0.004 ‘Gender’) + (0.318 ‘Physical work ability’) + (0.373 ‘Mental/emotional work ability’). Gender was coded as zero for men and one for women.

**Table 4:** Explained variance in appraised overall work ability by physical or/and mental/emotional work ability

	Overall work ability all ambulance workers		Overall work ability drivers		Overall work ability paramedics	
	*R* ^2^	Sig.	*R* ^2^	Sig.	*R* ^2^	Sig.
Physical work ability	0.374	0.000	0.397	0.000	0.360	0.000
Mental/emotional work ability	0.391	0.000	0.416	0.000	0.378	0.000
Together	0.483	0.000	0.507	0.000	0.470	0.000

## Discussion

Outcomes in ‘raised alertness and judgment ability’ and ‘emotional peak load’ explained statistically significant variability in appraised overall work ability. Outcomes in ‘job-specific physical abilities’ and ‘raised alertness and judgment ability’ explained statistically significant variability in physical work ability. Outcomes in ‘emotional peak load’ and ‘raised alertness and judgment ability’ explained significant variability in mental/emotional work ability. The only difference between occupations occurs for mental/emotional work ability. For drivers, ‘raised awareness and judgment ability’ explains most variance, while ‘emotional peak load’ outcomes explain most variance in paramedics’ mental/emotional work ability.

Physical and mental/emotional work ability explained almost half of the variance of appraised overall work ability. Physical and appraised mental/emotional work ability explained the same proportion of variance in overall work ability. The explained variance of overall work ability by either physical and mental/emotional work ability is almost four percent higher in drivers than in paramedics.

Although hypothesized, no significant differences were found between ambulance drivers and ambulance paramedics. Physical and mental/emotional work ability together explained only 3% more variance of overall work ability for ambulance drivers than for paramedics. It was expected that physical work ability would explain more variance than mental/emotional work ability. In contrast to studies by Klasan et al. (2013) and van de Vijfeijke et al. (2013), this study found that appraised mental/emotional work ability explained more variance of overall work ability than appraisal of physical work ability did. These differences can partly be explained by the fact that we included emotional work ability in the question on mental work ability. This might cause the shift to increased importance for appraised mental/emotional work ability. Another plausible explanation for the difference in findings could lie in the different type of occupations that were studied. Van de Vijfeijke et al. studied an internet panel, with unknown occupations. Klasan et al. studied all occupations at an Institute of Emergency Medicine and only found a significant relation between the physical domain and a lower work ability on follow-up. No significant relationship between the psychological domain and lower work ability on follow-up was found. The reason for this may lie in the fact that also nurses/medical technicians were included in the study population, with possible other work demands. Finally, the differences in results can be explained by the fact that the physical and mental/emotional aspects in both studies were not measured in a job-specific way as was in ours.

The WHS is a preventive strategy to monitor the health of ambulance workers and map signals that could lead to health problems and a loss of work ability. Participation in the WHS is restricted to ambulance workers actively working as ambulance driver or paramedic at the time. This selection bias causes little variation and high grades for the three work ability appraisal questions. Another possible source for selection bias is that participation in the WHS is voluntary, which can also lead to ambulance workers who have more complaints or think they are at risk, being more likely to participate.

One limitation of this study can be found in the fact that emotional work ability and mental work ability cannot be split to determine their separate contribution. Furthermore, our cross-sectional design could be regarded as a limitation. However, in this study we did not aim to find predictors for future low work ability and causal relationships, but merely how work ability relates to the job-specific health requirements in ambulance workers. Therefore, a cross-sectional design was appropriate.

A strength of the current study is that information about job-specific health requirements is gathered using job-specific instruments. By doing so, we assessed job requirements that are focused on ambulance work, while most studies on work ability focused on general aspects of health and work ability usually, in the general working population. We used the single item questions to assess work ability. The single question used to quickly assess the appraised work ability has sufficient convergent validity compared to the seven-item WAI-questionnaire (El Fassi et al. 2013). This method was suggested to be used during medical examinations in occupational healthcare because of its user-friendliness. The appraisal of work ability was chosen as our dependent variable to be able to gain insight in how job-specific requirement categories contribute to appraised general, physical and mental/emotional work ability.

To our knowledge, this is the first study reporting the association with appraised work ability and job-specific requirement categories in contrast to specific instruments. This approach was chosen to determine the association of different job-specific health requirement categories that are important for ambulance work and overcome the problem that separate instruments only assess a small part of a job requirement. By categorizing variables that affect work ability, insight was gained into what aspects are assessed as important job-specific requirements to be able to perform ambulance work in a safe way. By clustering variables, information was obtained on the important requirements that have to be met by ambulance workers to satisfactorily fulfill their work and cope with their demanding working conditions without impairing their health.

Other studies reported the relation between physical and mental health and work ability, whereas this study researched the association between job-specific requirements and work ability (Koolhaas et al. 2014; van de Vijfeijke et al. 2013). This creates an opportunity for occupational health professionals to put the different scores in job-specific health tests into perspective. Occupational physicians can start interventions where insufficient scores on important categories are observed, to improve work ability. However, insufficient scores on other categories should still be treated.

The results of this study suggest that in addition to the job-specific requirement categories, there are gender differences in the experience of work ability in ambulance workers. But although significant differences were found between men and women, this difference can be confounded by the division of gender between the occupations in ambulance work. Therefore, models have been developed for the entire group and separately for drivers and paramedics, and these were corrected for gender.

Appraised work ability is often used as a quick self-report measure to assess the work ability in working populations. In this study, while only 1.2% of the ambulance workers graded one aspect of their own work ability as insufficient, based on the tests the number of people scoring insufficient on one or more job-specific functional tests was 68%. This is indicative that the self-appraisal of work ability might be overrated compared to measurable job-specific health requirements. On the other hand, all outcomes together explain only 17% of the variance of appraised work ability which indicates that the appraisal of work ability includes factors other than these job-specific tests. Evidently, both appraised and measured work ability outcomes contribute in different ways to the assessment of work ability of ambulance workers.

In this study, the association of different categories of requirements with the appraisal of work ability was studied. Although not more than 17% of the variance can be explained, this study shows the relative importance of the contribution of each category per work ability outcome. ‘Raised alertness and judgment ability’ was significant and thus present in each model without being the most important aspect for physical and mental/emotional work ability. However, it was the most important category for overall work ability. This means that measuring the magnitude of work-related fatigue, mental complaints, alertness and sleepiness seems important to prevent a loss of overall work ability.
